# Health-related publications on people living in fragile states in the alert zone: a bibliometric analysis

**DOI:** 10.1186/s13033-020-00402-6

**Published:** 2020-08-27

**Authors:** Waleed M. Sweileh

**Affiliations:** Department of Physiology, Pharmacology/Toxicology, College of Medicine and Health Sciences, An-Najah National University, Nablus, Palestine

**Keywords:** Fragile states, Health, Infectious diseases, Mental health, Bibliometric analysis

## Abstract

**Background:**

Fragile states pose a global challenge. Assessing health research activity on people living in these states can help identify neglected health domains in fragile settings. The objective of the current study was to assess and describe health research activity on people living in fragile states in the alert zone.

**Method:**

A bibliometric method was applied using SciVerse Scopus. Research articles published on people in fragile states in the alert zone were retrieved and analyzed. The Fragile State Index (FSI) score was used for selection of states in the alert zone. The analysis was limited to 1 year; 2018.

**Results:**

The search query found 2299 research articles giving an average of 2 research articles per one million population per year in the selected fragile states. The number of research articles per one million population was not significantly correlated (p = 0.053; r = − 0.349) with FSI scores. However, it was significantly correlated with the extent of international research collaboration (p < 0.01, r = 065). Research on communicable diseases was the largest research domain (763 articles; 33.2%) followed by maternal/women’s health (430 articles; 18.7%), non-communicable diseases (291 articles; 12.7%), health system/policy (271 articles; 11.8%) and psychosocial and mental health (89; 3.9%). There were three research themes in the research domain of infectious diseases: HIV/AIDS; water-borne infectious diseases; and miscellaneous infectious diseases such as tuberculosis and malaria. The top ten cited articles were mainly on infectious diseases, particularly on malaria and Lassa fever. Of all the retrieved documents, 727 (31.6%) research articles appeared in national/regional journals while the remaining appeared in international journals. The World Health organization was the most active funding organization for research on fragile states. Top ten active institutions were mainly based in fragile states with the lowest FSI score, specifically Ethiopia, Uganda, Nigeria, and Pakistan.

**Conclusion:**

Research on fragile states was relatively low. Research on mental health and health system/policy should be encouraged. Collaboration and funding might help academic institutions in fragile states to make health problems in these countries more visible.

## Background

Fragile states have recently emerged as a key priority in the international development community for reasons related to human security, peacebuilding, and development [1, 2]. It is estimated that approximately more than one billion people are living in fragile states, mostly in low-income countries [3]. So far, there is no internationally-agreed definition of the term “fragile state” or fragility [4]. However, different international agencies agree that a fragile state is characterized by failure to meet its citizens’ basic daily demands. It is also agreed that without international support and engagement, a fragile state will continue to fall apart [3, 5, 6]. Fragile states need technical and financial support from the international community to face the challenges linked to fragility and to help these states achieve sustainable development goals [7].

Different international agencies have different definitions and criteria for the term “fragile states”. For example, the World Bank describes a country as ‘fragile’ if it (a) is eligible for assistance (i.e., a grant) from the International Development Association (IDA) (b) has had a UN peacekeeping mission in the last 3 years, and (c) has received a ‘governance’ score of less than 3.2 (as per the Country Policy and Institutional Assessment (CPIA) index of The World Bank) [8]. The list of fragile and conflict-affected situations (FCS) is released annually by the World Bank Group (WBG) and includes categorization of countries based on social fragility and severity of violence [9]. The United States Agency for International Development (USAID) defines the term “fragile states” as a broad range of failing, failed, and recovering states that are unable or unwilling to adequately assure the provision of security and basic services to a significant portion of their populations and where the legitimacy of the governments is in question [10]. The Organization for Economic Co-operation and Development (OECD) defines a fragile state as “a fragile region or state has weak capacities to carry out basic governance functions, and lacks the ability to develop mutually constructive relations with society. Fragile regions or states are also more vulnerable to internal or external shocks such as economic crisis or natural disaster” [11]. According to the OECD, fragility refers to a wide array of situations: countries in crisis, countries at war, reconstruction context, humanitarian and natural crises, and situations of extreme poverty [12, 13]. Other international agencies such as European Union, G7, Swiss Agency for Development and Cooperation (SDC), African Development Bank, and others have developed their definition and criteria for the term “fragile states”.

At the health level, people living in fragile states suffer from poverty, malnutrition, violence, high child and maternal mortality, psychological trauma, poor health systems with inadequate staff and medication, poor infrastructure, and high risk of infectious diseases [14, 15]. Strengthening and building resilient health systems in fragile states is important for global health security agendas since serious disease outbreaks in fragile states might affect the whole world [16]. For example, fragile states are poorly equipped to confront an outbreak of COVID-19 and therefore, without international help, the global health security is placed at high risk. Fragile states are major sources of mass migration leading to refugee crisis and disruption of health systems of the receiving countries [17, 18]. Leaving fragile states behind will create dangerous pockets of serious infectious diseases that might affect the whole world. The World Health Organization (WHO) released the top ten health threats in 2019 that included one about vulnerable and fragile settings where more than 1.6 billion people live in places with challenges such as drought, famine, conflict, population displacement, and weak health services [19].

Building a strong health system in any country requires strong leadership and governance, good information on health challenges facing the nation, health financing to reduce inequalities and ensure universal health coverage, human resources and health workforce, availability of sound quality essential medicines and technologies used in diagnostic procedures, and finally, the availability of primary healthcare system capable of providing cost-effective and close health service delivery [20]. Investment of high-income countries in health infrastructure and research capacity building in fragile states is believed to strengthen the national health system, global health security [21, 22], and limit international violence and terrorism [15, 23]. International engagement in health research in fragile states will provide detailed information on health challenges and health situation in the country. Such information is needed for strong leadership and governance of the health system. Building a strong state requires strengthening the health system and health research capabilities in that state. Fragile states have weak and broken health systems with limited human resources, limited research funding, and limited infrastructure needed to investigate the health situation and expose serious urgent gaps to the international community to recruit technical and financial support toward these urgent health gaps.

The Fragile States Index (FSI) is a tool developed to measure fragility based on 12 indicators that measure cohesion, economic, social, and political vulnerability of any country [24]. The primary purpose of FSI is to assess the vulnerability of all sovereign states that are members of the United Nations which currently omits Taiwan, Kosovo, Western Sahara, Northern Cyprus, and the Palestinian Territories. The FSI uses a spectrum of categories labelled *sustainable,* *stable,* *warning, and* *alert* to categorize and rank states. The score for FSI ranges from 0 to 120. Countries with a score above 90 are termed the “alert” group. Countries with FSI score from 60.0 to 89.9 are termed “warning”. Countries with FSI score from 30.0 to 59.9 are termed “stable”. Finally, countries with FSI score from 0.0 to 29.9 are termed “sustainable”.

The objective of the current study was to carry out a bibliometric analysis of health-related articles published in 2018 on people living in fragile states in the alert zone (i.e. FSI > 90). The objective of the current study was not to measure research output by authors affiliated with fragile states in the alert zone. Rather, the objective was to analyze research activity on health-related issues on people living in the fragile states regardless of the country affiliation of the authors. Furthermore, the current study was not meant to map the term “fragile state” in the literature. The analysis aimed to shed light on volume and research domains, the status of research collaboration, and key players in publishing articles on people living in countries described as fragile states and ranked in the alert zone. A literature search using Google Scholar showed that several research articles have been published on the health status of certain countries in the alert zone [25, 26]. However, none was carried out to map important health issues of people living in fragile states in the alert zone. The current study will endorse the ambitions of international key players in shedding light on the topic of fragility and fragile states as an essential element of global peace and security if we aim to reach a better world [12]. At the scientific level, the current study will serve (1) research and health institutions interested in global health security to better assess research on fragile states [27]; (2) international health agencies to allocate research gaps in these fragile states and tailor health support and health aids based on the identified gaps; (3) international funding agencies to strengthen health systems in most neglected health aspects [28, 29]; and (4) create and encourage research collaboration with colleagues in fragile states to rebuild the health research capabilities [30].

## Method

### Database used

The database used to accomplish the objective of the current study was SciVerse Scopus. The choice of Scopus was made because it is larger and more comprehensive than Web of Science (WoS) and PubMed [31]. Scopus has approximately 23,000 indexed peer-reviewed journals in all scientific disciplines compared to approximately 13,000 indexed peer-reviewed journals in WoS. Scopus has been used as a tool in many previously published bibliometric studies [32, 33].

### Search strategy

The search strategy was built to achieve the objective of the current study. Therefore, the focus in the search stagey was on two items: (1) fragile states in the alert zone and (2) health-related publications. The approach used to achieve the objective was based on the following six steps (Additional file 1):

1. Fragile states selected to be part of the study were those in the alert zone (i.e. FSI > 90) according to the FSI score published in 2019 [24]. The number of countries in the alert zone was 31 and most of them were in the African and Eastern Mediterranean regions. The range of FSI scores was from 113.5 for Yemen to 90.1 for Mauritania. All countries in the 2019 alert zone had been in this zone for at least ten times in the past 15 years. The names of the thirty-one fragile states in the alert zone were entered in the advanced search of the Scopus search tool. The names, and its derivatives, of the fragile states were listed in the title search to make sure that the study was carried out in the vulnerable setting of the fragile state. The search strategy was not designed to retrieve documents published by authors from the alert countries. We were interested in documents discussing health issues in the fragile states regardless of the affiliation of the author(s) and that is why we used the title search strategy rather than the affiliation strategy.
2. Exclude false-positive documents such as:
  - Documents on “aspergillus Niger” or “Sudan dye” or “guinea pigs” which might be mistakenly counted as the country name for Niger or Sudan or Guinea.
  - Documents about refugees or migrants living outside their countries because the focus of the current study was on the health status of people living in the fragile states and not refugees living in Europe or North America or Australia.
  - Documents on non-human health. Therefore, documents about dogs, horses, and plant diseases were excluded.
  - Documents on U.S soldiers deployed in Iraq or Afghanistan were also excluded.
3. Limit the retrieved documents to research articles. Therefore, reviews, letters, books, notes and editorials were all excluded. We were interested only in research articles since they represent true research.
4. Documents in all subject areas (EXCEPT that in the subject area of medicine) were excluded. Therefore, documents in subjects’ areas such as agriculture, environment, social science, humanities, chemistry, physics, mathematics, astronomy, arts, economics, business, education, and biomedical sciences (basic microbiology, immunology, pharmacology, biochemistry, and molecular biology) were excluded. Documents in the basic biomedical sciences were excluded because the major interest in the current study was on health publications relevant to the public health where immediate intervention can be of value to the people living in fragile states.
5. Limit study period to 1 year; 2018. The study period was limited to 1 year for two reasons. First, the choice of 2018 was made to avoid bias. The list of 31 fragile states in the alert zone was published in 2019 based on data available from these countries in the years before. Therefore, the selection of 2018 as the year of study will create less error in data analysis. Second, the list of the fragile states in the alert zone is not the same across all the years. Therefore, applying the search strategy over a longer period will create an error due to the changing names of countries in the alert zone. The FSI score for the same country varies each year based on the political and economic variations [24].
6. Limit the retrieved documents to those published in English.

### Validation of search strategy

The search strategy was validated to ensure minimum irrelevant and maximum relevant documents. The validation method applied was similar to that used in the previously published studies [34]. In short, the top 200 cited documents in the retrieved literature were reviewed to make sure that all are within the human health domain and were carried out in the listed fragile states. Whenever a false-positive result was found, the search strategy was changed to eliminate the false-positive results. The author kept excluding irrelevant keywords until the top 200 cited documents were free of any false-positive results. However, this step alone is inadequate for validation and a second step was implemented to make sure that the search strategy retrieved the maximum number of relevant documents. The second step utilized the number of publications authored by each of the top ten active authors and compared it with the number of publications by the same authors obtained from their scientific profile in Scopus. The result of the *Pearson correlation test* was significant with higher correlation value (r > 95%) suggesting that there were minimum false-negative results. The process of the validation was carried by W.S, the main author of the manuscript.

### Data export

The retrieved data in Scopus was exported to Microsoft Excel for presentation. The exported data included: annual number of publications, names of journals publishing the retrieved documents, and institutions publishing the retrieved documents, names of funding agencies. For each variable, only the top active ten was presented in the manuscript. For example, the top ten active journals and institutions were presented. All analysis and data export were carried out on the same day (January 10, 2020) to avoid misinterpretation.

### Research domains

The retrieved documents were analyzed to find the number of documents on infectious diseases, health system, psychosocial and mental health, women/maternal health, and non-communicable diseases. The number for each theme was obtained by adding specific keywords after the main search query. For example, in case of the health system research theme we used the following keywords: title (“health system” or “health policy” or “health service*” or “access to medicine*” or “access to medication*” or “access to health*” or “barrier* to health*” or “health plan*” or “health* facilit*” or “health insurance” or “medical insurance” or “health strategy” or “health* preparedness” or “health coverage” or “health regulation*” or “medical system” or “sanitation system” or “medical profession” or “nursing satisfaction” or “medical law” or “health plan*” or “medical profession” or “health profession” or “operating room” or “surgical facility” or “surgical care” or “surg* theater”) or journal name (“health system” or “health service” or “health policy”)). Additional file 2 shows the search query and keywords used to retrieve the overall documents and documents in each various research domains. Validation of the search query for each research domain was the same applied for the overall search query (absence of false-positive in top-cited documents and correlation test for active authors) and was carried out by W.S, the main author of the manuscript. In bibliometric studies, false-positive results or documents that did not meet the criteria (health field and name of the fragile state present in the title) is immediately excluded by adjusting the search query. This is different from the case of the systematic review where a third author needs to judge whenever two raters differ regarding the suitability of a document. Also, in the case of bibliometric studies, one scientific database is used and therefore no duplicates were found as in the case of systematic reviews.

### Graphics and mapping

Contribution of various countries to the retrieved documents was visualized using network visualization map. In the map, the larger the node size, the greater the contribution of that country. Similar node color is an indication of similar research interest. International research collaboration among active countries was visualized using VOSviewer [35]. In the map, the thickness of the connecting line between any two countries is called link strength which is proportional to the strength of research collaboration. The greater the thickness, the stronger the research collaboration. The map also gives the total strength of research collaboration (Total link strength = TLS) which is an indicator of the overall research collaboration for each country relative to other countries.

### Research themes

The retrieved documents were mapped for most frequent terms in the titles and abstracts of the retrieved documents. The clusters appearing in the map represent major research themes in the retrieved documents. Terms with similar color are inter-related and considered a research theme. The map was created using VOSviewer for terms with a minimum of ten occurrences.

### Geographic distribution of the retrieved articles

The retrieved articles were analyzed based on geographic distribution. The WHO world region classification was adopted: the region of the Americas, the European region, the Western Pacific region, the African region, the Eastern Mediterranean region, and the South-East Asia region. The number of articles for each region was found by combining the overall research query with the list of all countries for each region. This methodology was obtained from previously published studies [36].

### Scientific quality of publications

The quality or impact of the journal was measured using the quartile ranking obtained from the Scimago Journal Rank. Journals in the Q1 rank are considered to have the highest impact while those in the Q4 had the least impact.

### Quality assessment

For the finalized search query, the author consulted with two independent external researchers to check the content of randomly selected twenty articles from the retrieved literature. For each researcher, an endnote file of the retrieved documents was sent and the consultant was asked to pick randomly at least ten articles to check for the content. The feedback from the consultants positively confirmed that the content of the randomly selected articles matched the search criteria of being in the health field and on one of the investigated fragile states. The same approach was applied to check for quality assessment of the retrieved documents for each research domain. However, in the feedback regarding research domains, the consultants argued that some articles could fit into more than one domain. Since the current study was mainly quantitative, articles which fit into more than one domain were counted in each domain creating a certain overlap in the results. The positive agreement of the consultants with the finalized search query adds up to the validity of the search query.

## Results

### Volume and research productivity

The search query found 2299 research articles. A total of 1607 (69.9%) research articles were funded. The WHO ranked first (69 articles; 3.0%) as a funding agency. The total number of the combined populations across the thirty-one fragile states was 1152 million. Therefore, the number of retrieved articles was equivalent to 2 research articles per one million population. The retrieved articles received 3660 citations, a mean of 1.6 citations per document per year. Additional file 3 shows the list of thirty-one fragile states with the number of research articles published on each fragile state with the corresponding number of research articles per one million population. The number of research articles per one million for each country was not significantly correlated (p = 0.053; r = − 0.349) with FSI scores. However, there was a general trend of lesser number of publications in countries with higher FSI score. The country which had the highest number of articles per one million population was Uganda followed by Kenya and the Republic of Congo with 6.7, 5.7, and 5.6 research articles per one million respectively. On the other hand, North Korea and Congo D. R had the least number of publications with 0.1 and 0.4 research articles per one million population. When the same search strategy was applied to Qatar (FSI = 43.7), the result was 250 publications in 2018, equivalent to 96 research articles per one million population. For Kuwait (FSI = 50.9), it was 77.4 research articles per one million population. For Bahrain (FSI = 63.9), it was 65.3 research articles per one million population.

### Research domains

Analysis of the retrieved articles showed that 763 (33.2%) were within the domain of infectious diseases, 430 (18.7%) were within maternal/women’s health domain, 291 (12.7%) were within the domain of non-communicable diseases (including nutrition disorders), 271 (11.8%) were within the domain of health system/policy, and 89 (3.9%) were on psychosocial and mental health domain (Table 1). The remaining were in miscellaneous subjects as complementary/alternative medicine, chemical weapons in Syria, and general public/environmental health.

**Table 1:** Research domains in the retrieved articles

Research domain	Number of articles^a^	% (N = 2299)
Infectious diseases	763	33.2
Maternal and women’s health	430	18.7
Non-Communicable diseases (NCD)	291	12.7
Health system/policy	271	11.8
Psychosocial and mental health	89	3.9

^a^There is a possibility of overlap, particularly between NCD and mental health domains

### Research themes

**Fig. 1:**
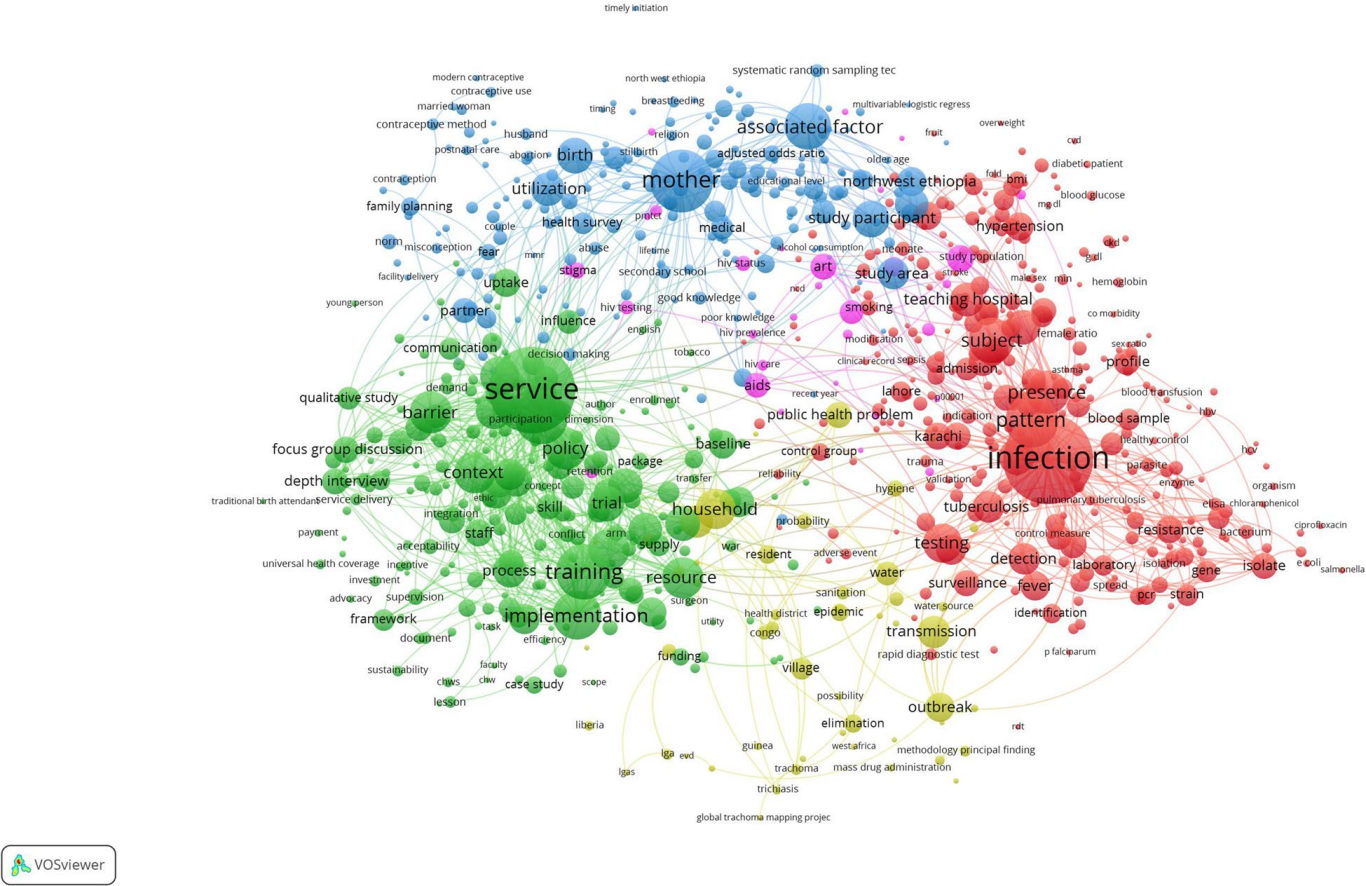
Network visualization of terms in the titles/abstracts of the retrieved articles with a minimum occurrence of 10 times. Terms with similar color represent closely related topic and exist in one cluster and is considered a research theme. The map had five research themes

Network visualization of terms in titles/abstracts with minimum occurrences of 10 yielded 832 terms that existed in five clusters representing five main different research themes (Fig. 1). HIV/AIDS constituted a separate research theme (pink cluster). Drinking water and hygiene constituted a research theme that included research on certain infectious diseases such as cholera, trachoma, onchocerciasis, and other tropical diseases (yellow cluster). The third cluster (green) focused on health services, access and barriers to health services, health system, health policy, hospital staff, resources, and training. The fourth cluster (red) focused on the epidemiology of infectious diseases (e.g. tuberculosis, hepatitis, measles, malaria) and non-communicable diseases (e.g. hypertension, diabetes mellitus, cardiovascular diseases, and chronic kidney diseases). The last cluster (light blue) focused on maternal, reproductive, women, and child health.

### Top ten cited research articles

The list of top ten cited research articles included nine about infectious diseases and one about stroke. Four of the top ten cited research articles discussed malaria and two discussed Lassa fever. The other discussed infections such as Ebola, Cholera, and Onchocerciasis. The top ten cited articles were published in high impact journals, particularly *The Lancet* [37–46].

### Top ten active journals

Table 2 shows the names of the top ten active journals that have been involved in publishing the retrieved documents. The top ten journals published 642 (27.9%) articles. Two of the top ten active journals were within the scope of infectious diseases, one was in the field of maternal health, one was in the field of health services, and the remaining were in the field of general medicine/public health. Four of the top ten active journals were national or regional journals while the remaining were international journals. The national/regional journals were *Pan African Medical Journal, African Health Sciences, Ethiopian Journal of Health Sciences, and Journal of the Pakistan Medical Association*. The journal that published the most was the *Pan African Medical Journal* (104 articles; 4.5%). All international journals in the top ten active list ranked Q1 while the national/regional journals ranked in the second or third quartile. Of all the retrieved documents, 727 (31.6%) articles were published in national/regional journals while the remaining were published in international journals.

**Table 2:** Top ten active journals in publishing health–related research articles on fragile states

Rank	Journal	Frequency	% (N = 2299)	Subject area	Journal rank	Country of the journal
1	Pan African medical journal	104	4.5	Medicine (miscellaneous)	Q3	Kenya
2	BMC public health	93	4.0	Medicine (Public Health, Environmental and Occupational Health)	Q1	UK
3	BMC Pregnancy and childbirth	71	3.1	Medicine (Obstetrics and Gynecology)	Q1	UK
4	BMC infectious diseases	67	2.9	Medicine (Infectious Diseases)	Q1	UK
5	Journal of the Pakistan medical association	64	2.8	Medicine (miscellaneous)	Q3	Pakistan
6	Plos neglected tropical diseases	57	2.5	Medicine (Infectious Diseases Public Health, Environmental and Occupational Health)	Q1	USA
7	Ethiopian journal of health sciences	53	2.3	Medicine (miscellaneous)	Q3	Ethiopia
8	BMC health services research	48	2.1	Medicine (Health Policy)	Q1	UK
9	BMJ open	46	2.0	Medicine (miscellaneous)	Q1	UK
10	African health sciences	39	1.7	Medicine (miscellaneous)	Q2	Uganda

Q = quartile. Q1 is the highest and Q4 is the lowest rank. The rank was obtained from Scimago Journal Rank (https://www.scimagojr.com/)

### Active countries and international research collaboration

**Fig. 2:**
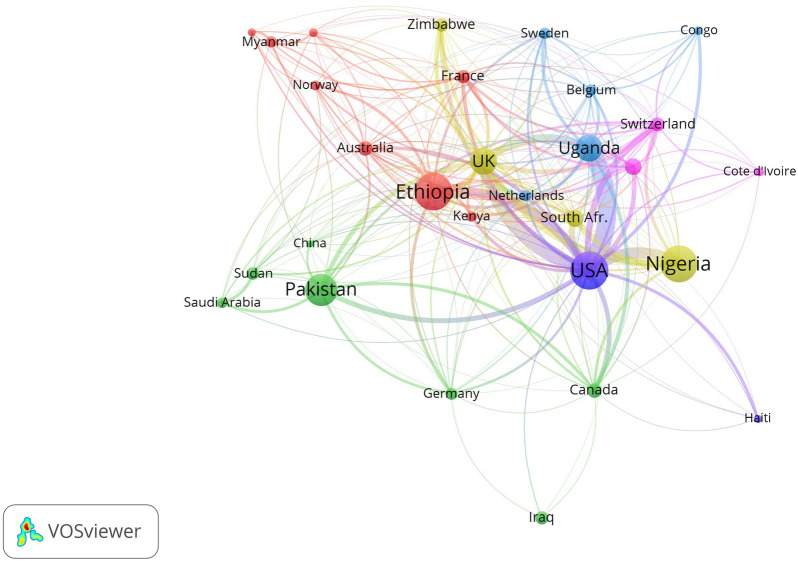
Network visualization of countries with a minimum contribution of 20 articles. The node size is proportional to the number of articles produced by that country. Similar node color indicated related research interest

Authors from 149 countries participated in publishing the retrieved articles. However, 47 (31.5%) countries contributed to a minimum of 10 publications and 29 (19.5%) countries contributed to a minimum of 20 publications in the retrieved literature. Network visualization of the 29 countries showed that six countries made a noticeable contribution to the retrieved countries (Fig. 2). Countries with larger node size had a higher volume of contribution. The six countries were the USA, Ethiopia, Pakistan, Nigeria, the UK, and Uganda. The remaining 23 countries in the map were characterized by small node size and distant location from the center indicative of relatively low research contribution and inadequate research collaboration.

**Fig. 3:**
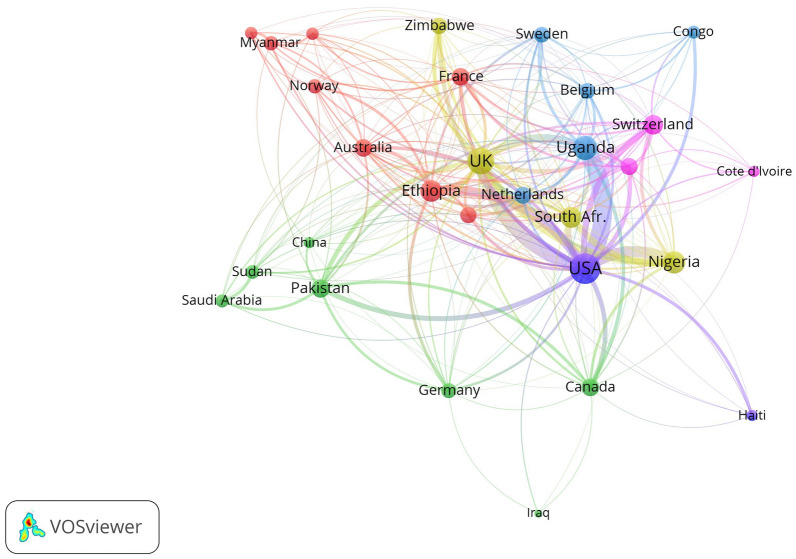
Network visualization of international research collaboration for countries with a minimum contribution of 20 articles. The thickness of any connecting line is a measure of the strength of research collaboration. Countries with large node size has large number of articles with international researchers while countries with small node size and located at the boundaries of the map have limited international research collaboration

International research collaboration was visualized for the 29 countries with a minimum of twenty publications (Fig. 3). The strongest research collaboration was between the USA and Uganda (TLS = 136) followed by that between the USA and Nigeria (TLS = 74). The extent of international research collaboration for each country in the fragile state list was presented as TLS in Table 1. There was a strong positive and significant correlation between the number of publications per one million for each fragile state and the strength of international research collaboration (Pearson correlation test: r = 0.65, p < 0.01).

### Research activity by world region

Analysis of the retrieved documents based on WHO world regions is shown in Table 3. The African region included 20 fragile states. Countries in the African region contributed to approximately two-thirds (1496; 65.1%) of the retrieved documents. The Eastern Mediterranean region included eight fragile states and contributed to 546 (23.7%) documents. The contribution of countries in the region of the Americas and the European region was almost equal. The contribution of the South-East Asia region and the Western Pacific region was the least.

**Table 3:** Geographic distribution of the fragile stated investigated the contribution of each region to the retrieved literature

WHO region	Number of fragile states in the region	List of fragile states	Number (%) of publications on health related issues in fragile states
The region of the Americas	1	Haiti	561 (24.4)
The European region	0	–	554 (23.7)
The African region	20	Burundi, Cameroon, Central African Republic, Chad, D.R of the Congo, Eritrea, Ethiopia, Guinea, Guinea-Bissau, Mali, Mauritania, Niger, Nigeria, Uganda, Zimbabwe, Chad, Kenya, Liberia, Cote d’Ivoire (Ivory Coast), Republic of Congo.	1496 (65.1)
The Eastern Mediterranean region	8	Yemen, Syria, Iraq, Pakistan, Afghanistan, Sudan, Somalia, Libya	546 (23.7)
The South-East Asia region	2	Myanmar, North Korea	81 (3.5)
The Western-Pacific region	0	–	145 (6.3)

### Top ten active institutions

The top ten active institutions/organizations involved in publishing the retrieved documents were shown in Table 4. The WHO and the *London School of Hygiene & Tropical Medicine* were the only institutions/organizations not based in the fragile states while the remaining eight were based in the fragile states particularly in Ethiopia, Nigeria, Uganda, and Pakistan.

**Table 4:** Top ten active institutions/organizations involved in publishing health–related research articles on fragile states

Rank	Institution/Organization	Number of publications	Frequency (N = 2299)	Country
1	Makerere University	152	6.6	Uganda
2	Addis Ababa University	144	6.3	Ethiopia
3	University of Gondar	113	4.9	Ethiopia
4	London School of Hygiene & Tropical Medicine	97	4.2	UK
5	University of Nigeria	76	3.3	Nigeria
5	Organisation Mondiale de la Santé	76	3.3	WHO
7	University of Ibadan	69	3.0	Nigeria
8	Bahar Dar University	56	2.4	Ethiopia
8	Jimma University	56	2.4	Ethiopia
10	The Aga Khan University	52	2.3	Pakistan

### Hypothetical growth of publications

When the search query was applied in the year 2017 and 2019, the research output was 2144 and 2701 documents respectively. Therefore, the research output in 2018 represented approximately 7% increase in the number of publications relative to 2017 while the year 2019 represented an increase of approximately 17.5% in the number of documents compared to the year 2018. These numbers might not be an accurate representation of the actual research output from the fragile states investigated in the current study because the list of the fragile states in the year 2018 might not be the same as those in 2017 or 2020.

## Discussion

The current study aimed to describe and analyze health-related publication on people living in fragile states listed in the alert zone based on the FSI scores. The vulnerable and fragile setting is considered by the WHO as one of the top ten global health threats [19]. Therefore, shedding light on health research on people living in fragile states is justifiable and in support of the WHO vision to tighten global health security by minimizing or eradicating sources of health threat.

The current study showed a relatively limited volume of health-related publications on people living in the selected fragile states when compared to other countries. This might reflect a lack of adequate international interest on the health situation in these countries. It is also possible that security problems hinder researchers from developed countries to participate in health studies on fragile states. The research capacities in fragile states might also be limited by the lack of adequate infrastructure or human resources which further limit potential research collaboration with researchers from developed countries. Fragile states might lack the infrastructure needed to educate and train health professionals to develop medical skills and research capacities [47, 48]. Health research is needed to uncover all health aspects in fragile states and guide the international community in their future intervention.

The current study showed a limited number of publications on psychosocial and mental health field on people living in the fragile states. Poverty, substance abuse, and violence against women are associated with fragile states and vulnerable settings [49]. A recent systematic review suggested that both depression and post-traumatic stress disorder were highly prevalent in war survivors who stayed in areas of conflict [50–52]. Even refugees who live abroad continue to suffer mental health problems due to the horrific sciences and scary journey to safe places [53]. The state of war and conflict reflects negatively on the mental health of children and women [54]. Therefore, upgrading mental health services and directing research toward mental health problems in children, women, and elderly are highly needed [54]. Furthermore, interventions by the national health authorities or international health organizations should focus on these vulnerable groups using cost-effective and sharing techniques [55–57]. The limited number of publications on psychosocial and mental health reflects weak mental health services and limited mental health experts in the selected fragile states. This seems to be common not only in fragile states but also in low- and middle-income countries. For example, the African region has 1.4 mental health workers per 100,000 people, compared with a global average of 9.0 per 100,000. The same weakness applies to other mental health services [58].

The current study also showed that research on the health policy/system constituted less than 12% of the retrieved literature despite that the health system in fragile states is poorly functioning. A developed and resilient health system in any country is the guarantee for minimizing health-related aspects of outbreaks or natural disasters [27, 59]. In conflict and post-conflict areas, health facilities and health workers might come under attack which further weakens the health systems. An example of the attack on health facilities and health workers has been reported in Syria [60, 61]. The attack on health facilities is an important cause of limited health human resources and weak health systems in these fragile states [62]. The Ebola crisis in certain areas in Africa is also a strong example of how weak health systems in fragile countries could not face and contain a serious disaster or infectious outbreak [63, 64]. Research on health system/services/policies in fragile countries might help international and national funding authorities to fill the gaps and build sustainable health systems in fragile states [65, 66]. The Health Systems Global Conference series had emphasized the importance of research on resilience and fragility as a lesson learned from the Ebola crisis. Achieving a strong and resilient health system in fragile states is an important step in building a strong state and major contributor to state legitimacy. Furthermore, building strong health systems and services in fragile states will minimize maternal death and will improve health services to mothers and patients with chronic diseases. Unfortunately, most of the international aids to fragile states is limited to humanitarian relief which does not advance either health systems development or state legitimacy. This might explain the persistent nature of conflicts in Syria, Libya, Somalia, Yemen, Congo, and many other countries in Africa [67, 68]. Strengthening health systems in fragile states should be a priority in the global health agenda and for international health organizations. Humanitarian relief to fragile states and economic investments in fragile states are mostly driven by political agendas rather than a global health security agenda. The current study also showed that the majority of fragile states lack adequate international research collaboration with high-income countries. A recent study on health system research in fragile and conflict-affected states indicated that collaboration is a key challenge to conducting health research in fragile states. Reasons cited for this were the presence of many different actors in health systems strengthening efforts and the language barriers [69].

The current study showed that the volume of research on maternal/women’s health was next to that of infectious diseases. According to the United Nation Population Fund, in 2015 the maternal mortality in countries affected by humanitarian crisis due to conflict was 417 per 100,000 live births, which is 1.9 times higher than the global estimate of 216 [70]. Closing the gap requires the involvement of the private sector as well as international organizations to address maternal and neonatal health services in fragile states. A study on maternal and neonatal health in fragile and conflict-affected areas in Asia and the Middle-East indicated that poor maternal and neonatal health services is a leading contributor to the burden of maternal and neonatal ill health in Asia and Middle-East and additional resources and policy attention are needed to address key barriers to effective maternal and neonatal care [71]. A study published recently in *The Lancet* about women and children in conflict areas in Africa concluded that deaths of young women in sub-Saharan Africa are exceptionally high relative to developed countries and effective approaches to prevent and mitigate the deleterious effects of armed conflicts on women and children should be a global priority [72]. Another study indicated that young people, including adolescents, continue to be a neglected group in humanitarian settings and more attention is needed to the sexual and adolescents health in humanitarian settings [73].

The lack of stable health systems, as well as lack of access to medications, is an important risk factor for various NCD such as hypertension, diabetes mellitus, and cardiovascular diseases in many fragile states. Unfortunately, the prevalence of hypertension is reportedly increasing in low- and middle-income countries. A study showed that approximately three-quarters of individuals with hypertension lived in low- and middle-income countries and that the prevalence of hypertension decreased by 2.6% in high-income countries but increased by 7.7% in low- and middle-income countries from 2000 to 2010 [74]. Most of the countries in the alert zone are classified within low- and middle-income countries and therefore, non-communicable diseases such as hypertension is expected to be a true health burden in these countries. A report by the WHO stated that over 30% of males and females have raised blood pressure in five fragile countries: Afghanistan, Central African Republic, Chad, Mali, and Somalia–all of which are of low-income status [75].

Research on infectious diseases had the largest number of publications. The current study showed most research interests were directed toward malaria and infections that had an immediate potential for a global outbreak such as Ebola or Lassa fever. Early detection of infectious disease outbreaks is important for global health security. A review article on fragile states recommended implementing infectious disease surveillance to enhance detection of outbreaks [76]. Research on other serious and common infection must be encouraged. Tuberculosis is a well-known infection in poor countries killing approximately 1.5 million people in 2018 [77]. The eradication of TB by 2030 requires the elimination of certain pockets in fragile states such as Pakistan, Afghanistan, and Nigeria [77, 78]. Helminthiasis, scabies, schistosomiasis, enteric bacterial and parasitic infections, as well as many other types of infections, are common in poor communities and research on these infections are also needed [79]. Therefore, more research efforts, funding, collaboration should be directed toward infectious diseases that do not pose an immediate threat to developed countries. Furthermore, research on fragile states with FSI scores above 100 (very high and high alert zone) such as Yemen need to be strengthened. Conflict in Yemen has generated several infectious disease outbreaks such as cholera and diphtheria which can cause mass fatalities [80–82]. The same applies to the situation in Syria where health teams and health facilities have been attacked in addition to the threat of chemical weapons [61, 83–85].

Three research themes within the retrieved literature focused on the infectious diseases: one on HIV/AIDS, a second one on water-borne diseases such as cholera, and the third one was on different types of infections including tuberculosis. One of the major issues in infectious diseases that threaten the spread of infectious diseases is the drop in immunization rates in children which might lead to outbreaks of measles, polio, and other diseases. For example, coverage for each vaccination was above 80% in Syria before the civil war in 2011 and dropped to 53% for measles and 41% for hepatitis B and DPT among 1-year-old [75]. The second important issue regarding infectious diseases in fragile states is that in conflict settings, especially in Africa, seven out of ten women are exposed to sexual violence and more than 50% of those women are most likely to develop HIV and transfer it to their babies [86]. The third issue regarding infectious diseases in fragile states is the poor water supply, poor sanitation system, and poor hygiene leading to cholera, trachoma, diarrhea, and other water-borne infectious diseases [87].

Certain fragile states had received a good number of publications. Most of these countries, e.g. Uganda, Nigeria, Pakistan, Congo, and Cameron had an FSI score below 100. Despite that, gaps in certain research domains have been found. For example, more research on psychosocial and mental health research domain is needed since the volume of research on this domain was the minimum. Another potential reason for the reasonable contribution of these fragile states is the presence of academic institution with medical facilities and health-related journals indexed in Scopus. Authors in these countries had a better chance than authors in other fragile states to disseminate their research observations in local journals. The international community needs to support these academic journals to make local research in fragile states more visible to international health and political communities.

The current study indicated that the African region had the highest contribution to the retrieved articles. This was not surprising given that the bulk of investigated fragile states were in the African region. Furthermore, the presence of a few medical journals indexed in Scopus and based in the African region helped increased the contribution of the African region relative to other world regions. Thirdly, the nature of problems facing the fragile states in the African region is also facing other stable countries in the African region such as South Africa. For example, HIV/AIDS, tuberculosis, malaria, trachoma, female genital mutilation, teen pregnancies, water security, climate change, and many other health-related problems are present in almost all African countries but with varying degrees [88].

## Limitation

This was a bibliometric analysis that used the FSI score for the inclusion of countries. However, the FSI score might not be the perfect indicator of fragility and therefore the results obtained in this study should be interpreted based on the methodology adopted to calculate the FSI score [89]. In the current study, we used the title search for all selected countries to find the volume and pattern of publications on people living in the selected fragile states. This methodology might not be a perfect one but it is the most feasible and the most accurate. Using affiliation strategy will retrieve a large number of publications that are irrelevant to people living in fragile states. Furthermore, using the title/abstract search strategy will also retrieve many false-positive results. Therefore, the approach adopted in the current study is the one with least false-positive results. Regardless of the pros and cons of the search methodology adopted, the current study was meant to draw attention to health status in fragile states through shedding light on research volume and research pattern on the selected fragile states. The goal was not to negatively criticize or expose weak research activity in this field. The goal was to promote research on this field as an important contributor to global health security.

## Conclusion

The volume of publications on fragile states was relatively low suggesting that people in these countries did not receive adequate attention from a research aspect. The largest research domain of interest on these countries was infectious diseases with emphasis on infections with a potential global threat. Psychosocial and mental health research domain was under-represented. The same applies to health system research. Research funding and collaboration to address these research domains are required.

## Supplementary information

**Additional file 1**. Health-related publications on fragile states in the alert zone: a bibliometric analysis. Flow diagram of study selection using Scopus database.

**Additional file 2.** Search query and keywords. Health-related publications on fragile states in the alert zone: a bibliometric analysis.

**Additional file 3.** List of thirty-one fragile states included in the study along with the number of publications per one million.

## Data Availability

All data presented in this manuscript are available on Scopus database using the search query listed in the methodology section.

## Abbreviations

WHO: World Health Organization
FSI: Fragile State Index
Q1: First Quartile
