# Supplementary material for: Health-related publications on people living in fragile states in the alert zone: a bibliometric analysis
Source: Int J Ment Health Syst. 2020 Aug 27;14:70. doi: 10.1186/s13033-020-00402-6 (PMC7450913; doi:10.1186/s13033-020-00402-6)
Supplement: Supplementary file 1 — Additional file 1. Health-related publications on fragile states in the alert zone: a bibliometric analysis. Flow diagram of study selection using Scopus database. [file 13033_2020_402_MOESM1_ESM.docx]

**Additional file 1**

**Health-related publications on fragile states in the alert zone: a bibliometric analysis**

Flow diagram of study selection using Scopus database

Number of documents on 31 fragile states listed in title search

**N= 307912**

Number of documents on fragile states after exclusion of false positive documents (e.g. documents on animals and documents with similar names to fragile states (e.g. "Sudan dye")

**N= 196541**

Limit to journal research articles

**N= 156271**

Exclude all subject areas EXCEPT "Medicine"

**N = 38556**

Limit to documents published on 2018

**N= 2323**

Limit to documents in English

**N = 2299**
