# Supplementary material for: Health-related publications on people living in fragile states in the alert zone: a bibliometric analysis
Source: Int J Ment Health Syst. 2020 Aug 27;14:70. doi: 10.1186/s13033-020-00402-6 (PMC7450913; doi:10.1186/s13033-020-00402-6)
Supplement: Supplementary file 2 — Additional file 2. Search query and keywords. Health-related publications on fragile states in the alert zone: a bibliometric analysis. [file 13033_2020_402_MOESM2_ESM.docx]

**Additional file 2**

**Search query and keywords**

**Health-related publications on fragile states in the alert zone: a bibliometric analysis**

| **Search query and keywords** | **Component** |
| --- | --- |
| (title ( yemen* or somali* or "south sudan*" or "syrian arab" or "syria*" or " democratic republic of the congo" or "congo d. r" or "african republic" or "chad" or "tchad" or sudan* or afg*anistan or afghani* or zimbabwe or guinea or haiti or iraq or nigeria or burundi or cameroon or eritrea* or niger or "guinea-b*" or uganda or mali or myanmar or ethiopia or pakistan* or "north korea" or "republic of the congo" or libya* or liberia or "cote d'ivoire" or "ivory coast" or mauritania* ) and not title-abs ( "service men" or "chad*s score" or pregabalin or "asper* niger" or pigs or "guinea pig*" or veteran* or army or soldier or "armed force*" or combat or hamster or deployment or dyes or "sudan iv" or *migrant or refugee or military or gulu or "sudan black b" or horses or animals or "sudan viruses" or "sudan ebola" or "sudan i" or cattle) and not srctitle ( "animal health" or "environmental research letters" or foraminiferal or evolution) and ( limit-to ( srctype,"j" ) ) and ( exclude ( doctype,"er" ) or exclude ( doctype,"le" ) or exclude ( doctype,"no" ) or exclude ( doctype,"ed" ) or exclude ( doctype,"tb" ) or exclude ( doctype,"re" ) or exclude ( doctype,"cp" ) or exclude ( doctype,"sh" ) or exclude ( doctype,"undefined" ) ) and ( exclude ( subjarea,"soci" ) or exclude ( subjarea,"agri" ) or exclude ( subjarea,"envi" ) or exclude ( subjarea,"bioc" ) or exclude ( subjarea,"eart" ) or exclude ( subjarea,"arts" ) or exclude ( subjarea,"busi" ) or exclude ( subjarea,"econ" ) or exclude ( subjarea,"immu" ) or exclude ( subjarea,"engi" ) or exclude ( subjarea,"phar" ) or exclude ( subjarea,"ener" ) or exclude ( subjarea,"nurs" ) or exclude ( subjarea,"mult" ) or exclude ( subjarea,"comp" ) or exclude ( subjarea,"psyc" ) or exclude ( subjarea,"vete" ) or exclude ( subjarea,"chem" ) or exclude ( subjarea,"heal" ) or exclude ( subjarea,"ceng" ) or exclude ( subjarea,"math" ) or exclude ( subjarea,"deci" ) or exclude ( subjarea,"phys" ) or exclude ( subjarea,"neur" ) or exclude ( subjarea,"mate" ) or exclude ( subjarea,"dent" ) ) and ( limit-to ( pubyear,2018) and ( limit-to ( language,"english" ) ) ) | **Overall search query** |
|  |  |
| srctitle (psychiatry or "mental health" or "substance abuse" or psychology or psychosis or schizophrenia) or title ("mental illness" or behavioural or emotional or "post-trumatic" or addiction or "substance abuse" or depression or depressive or suicide or psychology* or "mental health" or anxiety or "alcohol use" or "drug abuse" or bipolar or "mood disorder" or psychiatry or dementia or schizoph* or anti-depress* or mania or phobia or stigma or "social isolation") | **Psychosocial and mental health** |
| title("health system" or "health policy" or "health service*" or "access to medicine*" or "access to medication*" or "access to health*" or "barrier* to health*" or "health plan*" or "health* facilit*" or "health insurance" or "medical insurance" or "health strategy" or "health* preparedness" or "health coverage" or "health regulation*" or "medical system" or "sanitation system" or "medical profession" or "nursing satisfaction" or "medical law" or "health plan*" or "medical profession" or "health profession" or "operating room" or "surgical facility" or "surgical care" or surgery) or srctitle("health system" or "health service" or "health policy" or "surgery room" or "surg* theater") | **Health policy and systems** |
| srctitle ( infectious or infection or malaria or "tropical disease*" or "parasit*" or virology or "tropical medicine" or virus or hiv or virology or microbiology or vaccine* ) or title (di*rrhea or enteric or dysentery or "urinary tract infection*" or vaccine* or immuniz* or trachoma or plasmodium or "waterborne disease*" or "west nile virus" or ebola or babesiosis or "red tides" or hantavirus or hiv or "immunodef* virus" or "rift valley fever" or "h*emorrhagic fever" or helminth* or "mosquito borne" or "common cold" or amebia* or myiasis or ascariasis or pediculosis or scabies or toxoplasmosis or "parasite" or candida* or malaria or virus or tuberculosis or zika or herpes or "avian *flu*" or "swine *flu*" or helminth* or onchoce* or zoonotic or brucell* or pneumonia or schistos* or buruli or lyme or toxoplas* or leptospirosis or pediculosis or chagas or rabies or coronavirus or zoono* or "resp* tract infect*" or cytomegalo* or "tropical disease*" or measles or mumps or pneumonia or echinococcosis or meningitis or cholera or chikungunya or rubella or smallpox or filariasis or "vector-borne" or leprosy or poliomy* or taeniasis or cysticercosis or trypanos* or varicella or acinetobacter or strongyloid* or *infection or giardia or trypano* or "japanese enceph*" or hepatit* or "sars" or shigella or taenia or toxoplasm* or pneumonia or anthrax or cryptococcosis or rubella or plague or chikungunya or mers-cov or tularaemia or infection or infectious or dengue or "parasite" or "communicable diseases" or "neglected tropical diseases" or "yellow fever" or plague or arbovir* or infection or diarrh* or waterborne or trachoma or malaria or schistosomiasis or "typhoid fever" or amoebiasis or cholera or hepatitis or salmonell* or shigellosis or dengue or onchocerciasis or "japanese encephalitis" or scabies or conjunctivitis or "scrub typhus" or leptospirosis or "communicable diseases" or norovirus or cryptosporidium or "entamoeba histolytica" or giardia or shigella or salmonella or campylobacter or "escherichia coli" or "tick-borne" or "borrelia burgdorferi" or "encephalitis virus" or plasmodium or leptospirosis or kala-azar or virus or "water borne" or "air borne" or "food borne" or "vector borne" or plasmodium or anophole* or "rodent-borne" or hantavirus* or malaria or dengue or mosquito or cholera or lassa or contamination ) | **Infectious diseases** |
| srctitle ( heart or cardiac or renal or respiratory or neurology or kidney or nutrition) or title (diabet* or hypertens* or heart or cardiac or cardiovasc* or asthma or epilepsy or parkins* or neurology* or renal or kidney or stroke or rheumatoid or osteoarithritis or cerebrovasc* or "chronic disease*" or "autoimmune disease" or alzheimer or cancer or oncology or leukemia or tumor or "high blood pressure" or nutrition or malnutri* or obesity or "food *security" or an*emia or iron) | **Non-communicable diseases** |
| srctitle (pregnancy or women or maternal or contraception) or title (wom*n or pregnan* or maternal or neonat* or "teen marriage" or "reproductive health" or "sexual health" or childbirth or antenatal or perinatal or mother)) | **Maternal and women's health** |
