# Supplementary material for: Health-related publications on people living in fragile states in the alert zone: a bibliometric analysis
Source: Int J Ment Health Syst. 2020 Aug 27;14:70. doi: 10.1186/s13033-020-00402-6 (PMC7450913; doi:10.1186/s13033-020-00402-6)
Supplement: Supplementary file 3 — Additional file 3. List of thirty-one fragile states included in the study along with the number of publications per one million. [file 13033_2020_402_MOESM3_ESM.docx]

| **Rank** | **Country** | **FSI* score 2019** | **Number of publications** | **Number of populations in millions (2018)**** | **Number of Articles per one million** | **TLS#** |
| --- | --- | --- | --- | --- | --- | --- |
| **1** | **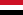**[**Yemen**](https://en.wikipedia.org/wiki/Yemen) | **113.5** | **26** | **29.2** | **0.9** | **35** |
| **2** | **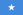**[**Somalia**](https://en.wikipedia.org/wiki/Somalia) | **112.3** | **17** | **15.4** | **1.1** | **5** |
| **3** | **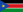**[**South Sudan**](https://en.wikipedia.org/wiki/South_Sudan) | **112.2** | **17** | **12.8** | **1.3** | **0** |
| **4** | **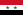**[**Syria**](https://en.wikipedia.org/wiki/Syria) | **111.5** | **24** | **17.1** | **1.4** | **2** |
| **5** | **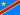**[**Democratic Republic of the Congo**](https://en.wikipedia.org/wiki/Democratic_Republic_of_the_Congo) | **110.2** | **28** | **86.8** | **0.3** | **22** |
| **6** | **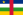**[**Central African Republic**](https://en.wikipedia.org/wiki/Central_African_Republic) | **108.9** | **10** | **5.5** | **1.8** | **9** |
| **7** | **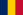**[**Chad**](https://en.wikipedia.org/wiki/Chad) | **108.5** | **7** | **15.7** | **0.4** | **16** |
| **8** | **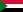**[**Sudan**](https://en.wikipedia.org/wiki/Sudan) | **108.0** | **67** | **42.2** | **1.6** | **75** |
| **9** | **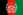**[**Afghanistan**](https://en.wikipedia.org/wiki/Afghanistan) | **105.0** | **24** | **32.2** | **0.7** | **25** |
| **10** | **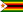**[**Zimbabwe**](https://en.wikipedia.org/wiki/Zimbabwe) | **99.5** | **68** | **15.2** | **4.5** | **97** |
| **11** | **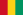**[**Guinea**](https://en.wikipedia.org/wiki/Guinea) | **99.4** | **36** | **12.2** | **3.0** | **32** |
| **12** | **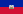**[**Haiti**](https://en.wikipedia.org/wiki/Haiti) | **99.3** | **32** | **11.6** | **2.8** | **27** |
| **13** | **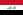**[**Iraq**](https://en.wikipedia.org/wiki/Iraq) | **99.1** | **68** | **39.2** | **1.7** | **12** |
| **14** | **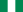**[**Nigeria**](https://en.wikipedia.org/wiki/Nigeria) | **98.5** | **492** | **201.0** | **2.4** | **278** |
| **15** | **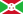**[**Burundi**](https://en.wikipedia.org/wiki/Burundi) | **98.2** | **7** | **11.0** | **0.6** | **7** |
| **16** | **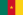**[**Cameroon**](https://en.wikipedia.org/wiki/Cameroon) | **97.0** | **89** | **25.9** | **3.4** | **121** |
| **17** | **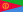**[**Eritrea**](https://en.wikipedia.org/wiki/Eritrea) | **96.4** | **5** | **3.5** | **1.4** | **11** |
| **18** | **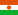**[**Niger**](https://en.wikipedia.org/wiki/Niger) | **96.2** | **15** | **23.3** | **0.6** | **16** |
| **19** | **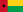**[**Guinea-Bissau**](https://en.wikipedia.org/wiki/Guinea-Bissau) | **95.5** | **1** | **1.6** | **0.6** | **0** |
| **20** | **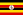**[**Uganda**](https://en.wikipedia.org/wiki/Uganda) | **95.3** | **266** | **40.0** | **6.7** | **369** |
| **21** | **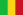**[**Mali**](https://en.wikipedia.org/wiki/Mali) | **94.1** | **20** | **20.0** | **1.0** | **34** |
| **22** | **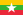**[**Myanmar**](https://en.wikipedia.org/wiki/Myanmar) | **94.3** | **61** | **54.3** | **1.1** | **69** |
| **23** | **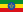**[**Ethiopia**](https://en.wikipedia.org/wiki/Ethiopia) | **94.2** | **498** | **98.7** | **5.0** | **241** |
| **23** | **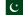**[**Pakistan**](https://en.wikipedia.org/wiki/Pakistan) | **94.2** | **371** | **218.3** | **1.7** | **159** |
| **25** | **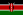**[**Kenya**](https://en.wikipedia.org/wiki/Kenya) | **93.5** | **273** | **47.6** | **5.7** | **94** |
| **26** | **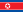**[**North Korea**](https://en.wikipedia.org/wiki/North_Korea) | **92.7** | **3** | **25.4** | **0.1** | **0** |
| **27** | **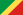**[**Republic of the Congo**](https://en.wikipedia.org/wiki/Republic_of_the_Congo) | **92.5** | **30** | **5.4** | **5.6** | **59** |
| **28** | **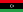**[**Libya**](https://en.wikipedia.org/wiki/Libya) | **92.2** | **8** | **6.8** | **1.2** | **1** |
| **29** | **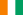**[**Cote d'Ivoire**](https://en.wikipedia.org/wiki/Ivory_Coast) | **92.1** | **28** | **25.8** | **1.1** | **37** |
| **30** | **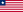**[**Liberia**](https://en.wikipedia.org/wiki/Liberia) | **90.2** | **22** | **4.5** | **4.9** | **39** |
| **31** | **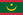**[**Mauritania**](https://en.wikipedia.org/wiki/Mauritania) | **90.1** | **4** | **4.1** | **1.0** | **2** |

**Additional file 3** List of thirty-one fragile states included in the study along with the number of publications per one million

***FSI = Fragile States Index Score obtained from** <https://fragilestatesindex.org/data/>

**** Population size was obtained from** <https://data.worldbank.org/country>

**#TLS = total link strength (index of international collaboration)**
